# Le mélanome malin: une tumeur rare des fosses nasales - à propos d'une série de 10 cas

**DOI:** 10.11604/pamj.2014.18.101.2778

**Published:** 2014-05-28

**Authors:** Amal Errachdi, Brice Nkoua Epala, Amal Asabbane, Naoual Kabbali, Mariem Hemmich, Tayeb Kebdani, Noureddine Benjaafar

**Affiliations:** 1 Pôle de Radiothérapie, Institut National d'Oncologie, CHU Ibn Sina, Rabat, Maroc

**Keywords:** Mélanome malin, fosses nasales, chirurgie, radiothérapie, pronostic, malignant melanoma, nasal cavity, surgery, radiotherapy, prognosis

## Abstract

Le mélanome malin des fosses nasales est une tumeur rare mais très agressive, de traitement complexe et de pronostic défavorable. Son traitement relève en principe d'une prise en charge essentiellement chirurgicale complétée par une radiothérapie. L'objectif de ce travail est de rapporter les caractéristiques cliniques, thérapeutiques et évolutives des mélanomes des fosses nasales. Nous avons analysé rétrospectivement 10 cas de mélanomes des fosses nasales suivis à l'institut national d'oncologie de Rabat. La rhinoscopie avec biopsie a permis la confirmation histologique du diagnostic de mélanome. Le bilan d'extension comprenait une tomodensitométrie ou imagerie par résonnance magnétique du massif facial, une radiographie thoracique et une échographie abdominale. Dans notre série, l’âge médian était de 67.5 ans, avec une prédominance féminine (7femmes et 3hommes). Le délai médian de découverte était de 6 mois. Deux patients étaient métastatiques d'emblée, et toutes les tumeurs étaient localement avancées au moment du diagnostic. Sept patients ont été opérés avec des limites chirurgicales envahies dans 2 cas et 3 patients étaient inopérables. 2 patients ont été irradiés après la chirurgie et 2 patients ont reçu une chimiothérapie arrêtée au moment de la progression. Deux patients ont récidivé après traitement, et un patient était en mauvais état général et a bénéficié uniquement de soins palliatifs. Tous les patients sont décédés avec un délai médian de survie de 12 mois. Le mélanome malin muqueux des fosses nasales, bien que rare, demeure une pathologie de pronostic défavorable et pose des problèmes de prise en charge.

## Introduction

Le mélanome malin des fosses nasales est une tumeur rare et agressive, de diagnostic difficile, d'aspect clinique aspécifique et sans facteur étiologique connu. Il nécessite un bilan pré thérapeutique précis (imagerie) et relève en principe d'une prise en charge essentiellement chirurgicale complétée par une radiothérapie dans un cadre multidisciplinaire afin d'optimiser au mieux le pronostic et d'assurer dans tous les cas une qualité de vie acceptable. Son pronostic reste sombre avec des taux de survie faibles, ne dépassant pas en moyenne 40% à 5ans [1]. L'objectif de ce travail et de préciser les caractéristiques cliniques, thérapeutiques et évolutives à travers une série rétrospective de 10 patients présentant un mélanome malin des fosses nasales traités à l'institut national d'oncologie de Rabat, avec revue de la littérature.

## Méthodes

Nous rapportons 10 cas de mélanomes malins des fosses nasales enregistrés à l'institut national d'oncologie sur une période de 10ans, de janvier2000 à Décembre2010. Seuls les patients ayant une confirmation histologique de mélanome malin primitif des fosses nasales ont été inclus. La confirmation histologique a été obtenue par biopsies sous rhinoscopie. Le bilan d'extension comprenait une tomodensitométrie (TDM) ou une imagerie par résonance magnétique (IRM) cérébrale, une Radiographie thoracique et une échographie abdominale.

## Résultats

Dans notre série l’âge médian était de 67.5 ans, avec une prédominance féminine (7 femmes et 3 hommes). Le délai médian de découverte était de 6 mois. L'obstruction nasale était le symptôme le plus fréquent, retrouvé chez 9 patients et des épisodes d’épistaxis étaient présents chez 2 patients. Chez une patiente, la maladie a été diagnostiquée à l'occasion d'une exophtalmie. L'histologie a été confirmée chez tous les patients (Tableau 1). L'immunohistochimie n'a été faite que chez 3 patients. A l'imagerie, une patiente avait un magma d'adénopathies cervicales de 7cm de diamètre, 2 patients étaient métastatiques d'emblée (l'un au niveau pulmonaire, l'autre au niveau hépatique), et toutes les tumeurs étaient localement avancées au moment du diagnostic. Sept patients ont été opérés: exérèse large avec des limites chirurgicales envahies dans 2 cas et trois patients étaient inopérables (tumeurs très localement avancées). Deux patients ont reçu une radiothérapie postopératoire à la dose de 60Gy, 2Gy par fraction en 6semaines chez un patient et 30Gy (3Gy par fraction) chez un patient. Parmi les patients inopérables, deux patients ont bénéficié de soins de support uniquement et une patiente a reçu 2 cycles de chimiothérapie à base de Cisplatine et interféron α avant de refuser de continuer le traitement. La patiente métastatique au niveau pulmonaire a reçu une chimiothérapie post-opératoire à base de dacarbazine, arrêtée au moment de la progression. Deux patients ont progressé après traitement et 6 patients ont récidivé. Il s'agissait de récidives locales dans deux cas, régionale et métastatique dans un cas et métastatique seule dans trois cas. Tous les patients sont décédés avec un délai médian de survie de 12 mois (Tableau 1).

**Tableau 1:** Caractéristiques cliniques et thérapeutiques des patients

**Sexe**	Femmes : 7 Homes: 3
**Age**	Moyenne: 67.7 ans Extrêmes: 58 – 80 ans
**Délai diagnostic médian**	4mois
**Symptomes**	Obstruction nasale : 9 patients Epistaxis : 2 patients Exophtalmie : 1 patiente Masse endonasale : 1 patient
**Stades**	Stade I: 7 Stade II: 1 Stade III: 2
**Traitement**	Chirurgie: 7 patients Radiothérapie adjuvante: 2 patients Chimiothérapie: Soins de support : 2 patients
**Survie**	Médiane: 12 mois Extrêmes : 8 – 30 mois

## Discussion

Le mélanome malin est une prolifération néoplasique maligne faite de cellules d'origine neuroectodermique de type mélanocyte avec ou sans pigment mélanique [1]. La localisation nasale est rare. Elle représente 0,5% des tumeurs de la sphère ORL, 0,4 à 0,7% de tous les mélanomes, environ 10% des mélanomes de la tête et du cou et 4% de l'ensemble des tumeurs malignes nasosinusiennes [2, 3]. Le premier cas de mélanome muqueux de la tête et du cou a été signalé par Weber en Allemagne en 1856 [4]. D′autres rapports de patients atteints de mélanome muqueux de la fosse nasale ont été publiés par la suite par Lucke [5] en 1869 et Viennois [6] en 1872. Plus tard, et en raison de la rareté de cette maladie, des études rétrospectives limitées ont été publiées et aucune étude prospective n'a été réalisée.

L’âge moyen de survenue est de 65 ans (extrêmes: 35-92 ans), plus élevé dans notre série (67.5ans). La répartition selon le sexe est variable. Vinel et al. [7] et Peralta et al. [8] ont noté une prédominance masculine, tandis que pour la série de Vallicioni et al. [9], comme pour notre série, il y avait une prédominance féminine. L'analyse de plus grandes séries de la littérature ne notait pas de prédominance de sexe [10]. Le septum nasal représente le site tumoral primitif dans 75% des formes endonasales, notamment sa partie antéroinférieure, suivi par la paroi externe (cornet inférieur, puis moyen) [11, 12]. Dans notre série, tous les patients avaient des tumeurs localement avancées au moment du diagnostic, le site tumoral primitif ne pouvant être défini avec précision. Le délai moyen entre les premiers symptômes et l'examen clinique est de 4 mois [13]. Ce délai était de 6mois pour notre série.

La symptomatologie clinique est peu spécifique, dominée par l'obstruction nasale, des épistaxis, des écoulements nasaux, ou plus rarement des douleurs. Les adénopathies sont exceptionnelles. L'endoscopie nasale permet un bilan topographique, une évaluation de l'extension tumorale et la pratique de biopsies dont l’étude anatomopathologique permet d’établir le diagnostic. Ce diagnostic est difficile du fait d'un double polymorphisme architectural et cytologique. Le diagnostic de certitude est apporté par les techniques d'immunohistochimie qui permettent de mettre en évidence les marqueurs de différenciation mélanocytaire et d’éliminer d'autres causes tumorales [14]. Nous disposons à l'heure actuelle de plusieurs marqueurs de différenciation dont la tyrosinase (marqueur le plus sensible pour les mélanomes nasosinusiens), le Mart-1/Melan-A, l'anti-gp100 (HMB-45), la protéine S100, la Microphtalmia transcription factor (MIFT), voire le PNL-2 [15]. L'imagerie est un élément fondamental dans la décision thérapeutique. L'IRM est l'examen le plus performant pour préciser l'extension locale dans les cavités sinusiennes et extranasosinusiennes, l'extension à l'orbite, à la fosse infra temporale et au sinus caverneux. Pour l'extension aux tissus mous de la face, la tomodensitométrie reste assez performante, mais l'IRM permet une étude plus fine [1]. Le bilan général de la maladie comprend une radiographie pulmonaire, une échographie abdominopelvienne et une scintigraphie osseuse [1]. Il n'existe pas de classification consensuelle pour les mélanomes des fosses nasales. La classification utilisée dans la plupart des séries est celle de Micheau et Marandas: stade I: local, stade II: régional, stade III: général [16]. Cette classification présente l'avantage de la simplicité mais elle reste peu précise. Les facteurs pronostiques ont été évalués dans plusieurs séries. En analyse uni variée, la taille tumorale, le stade, le sexe, la mélanodermie, la radiothérapie postopératoire et la rémission complète après le traitement initial étaient des facteurs pronostiques dans certaines études. En analyse multi variée, le jeune âge (moins de 50ans), et le stade précoce de la maladie ressortaient comme des facteurs de bon pronostic pour la survie globale [3, 17, 18].

Sur le plan thérapeutique, le traitement des mélanomes malins des fosses nasales, comme pour tous les mélanomes de la tête et du cou, est contesté. L'exérèse chirurgicale complète est le pilier du traitement de la maladie localisée avec ou sans envahissement ganglionnaire [19]. La radiothérapie postopératoire est généralement considérée pour la majorité des patients [17, 20], même si son rôle reste incertain [18, 21, 22]. Plusieurs études ont conclu que l′ajout de la radiothérapie à l′exérèse chirurgicale permettait un bénéfice de contrôle local même pour les petites tumeurs. Quelques études ont suggéré une amélioration de la survie chez les patients recevant une radiothérapie post opératoire [23–25]. Bien que la plupart des séries publiées n′ont pas retrouvé ce bénéfice en survie, de nombreux auteurs recommandent un traitement local agressif avec radiothérapie adjuvante ou de rattrapage pour les patients présentant des mélanomes des fosses nasales ou nasosinusiens [3, 20, 25, 26]. La radiothérapie hypo fractionnée est recommandée par la plupart des auteurs [1, 21]. Les doses sont variables ne sont pas bien établies, et varient selon les séries [21]. Certaines techniques comme la radiothérapie par modulation d'intensité ou la protonthérapie permettent d'optimiser la radiothérapie sans augmenter les effets secondaires [1, 21].

Malgré un traitement locorégional agressif, le décès survient chez la majorité des patients présentant des mélanomes des fosses nasales, suite à des rechutes métastatiques et les résultats pour ces patients dans presque toutes les études sont restés sombres. Les taux élevés de métastases soulignent l'intérêt des traitements systémiques. Malheureusement, la rareté des drogues efficaces limite l'amélioration du contrôle de la maladie systémique et de la survie [27]. Plusieurs drogues ont été utilisées telles que la dacarbazine, cisplatine, ranimustine et tamoxifène, mais leur rôle reste incertain. Actuellement, la Dacarbazine en mono-chimiothérapie avec une dose unique de 850-1000 mg/m2 ou une dose multiple: le protocole de 250 mg/m2 pendant 5 jours par cycle est considérée comme le traitement standard de première intention chez les patients avec un mélanome nasosinusien à un stade avancé [28, 29].

L′immunothérapie, à base de bacilles de Calmette et Guérin (BCG) le plus souvent, a été utilisée dans le traitement de certains cas isolés de mélanomes nasosinusiens, mais toujours en association avec d'autres thérapeutiques. Son efficacité devrait être évaluée par des études plus larges [28]. Même si ces thérapeutiques adjuvantes et palliatives sont largement utilisées, les résultats sur le contrôle local et la survie globale restent médiocres. Dans toutes les séries de la littérature, la survie globale à 5 ans après traitement des mélanomes des fosses nasales et de 20 à 40% et la médiane de survie est de 24mois (Tableau 2). Elle était de 12mois pour notre série. Les études de recherche actuelles s'orientent vers la thérapeutique moléculaire comme les facteurs de croissance, les inhibiteurs enzymatiques, les anti-angiogéniques et les drogues immunomodulatrices [29].

**Tableau 2:** Mélanomes malins des fosses nasales survie: données de la littérature

Auteurs	Résultats
Bonfils et al [11]	taux de survie à 3 ans de 25%
Vallicioni et al. [9] : (41 cas)	taux de survie à 5 ans de 40%
Kingdom et al [20]: (17 cas)	taux de survie 2 ans de 67%, à 5 ans de 20%
Castillo et al. [12]: (17 cas)	taux de survie à 2 ans de 52% ± 35%, à 4 ans de 26% ± 40%
Thompson et al. [3]: (115 cas)	taux de mortalité de 65,2% à 2,3 ans
Poissonet et al [10] : (12 cas)	taux de survie à 4 ans de de 26%
Hyodo et al [26] : (14cas)	taux de survie à 2 ans de 38.5%, à 5ans 20.5% et 10.2% à 10ans
Lund et al [23] : (72 cas)	taux de survie à 5 ans de 28%, à 10ans de 20%, survie médiane de 21mois

## Conclusion

Le mélanome malin muqueux des fosses nasales, est une pathologie rare, de pronostic défavorable qui pose des problèmes de prise en charge malgré les avancées thérapeutiques et techniques actuelles. Souvent diagnostiqué à un stade avancé et un grand âge, son profil évolutif est marqué par la fréquence des récidives locales et des métastases et les protocoles thérapeutiques peu codifiés. Le diagnostic précoce, un bilan locorégional et général précis et une prise en charge comprenant obligatoirement chirurgie et radiothérapie postopératoire hypo fractionnée pourraient contribuer à l'amélioration de la survie globale. Les thérapeutiques immunologiques (cytokines, interleukines, BCG) méritent des études randomisées.
